# Systemic therapy in metastatic renal cell carcinoma (mRCC): an evidence-based recommendation of the German interdisciplinary RCC guidelines group

**DOI:** 10.1007/s00345-022-04015-1

**Published:** 2022-05-13

**Authors:** V. Grünwald, L. Bergmann, B. Brehmer, B. Eberhardt, Karin Kastrati, T. Gauler, G. Gehbauer, J. Gschwend, M. Johannsen, T. Klotz, C. Protzel, M. Schenck, M. Staehler

**Affiliations:** Clinic for Medical Oncology and Clinic for Urology, University Hospital Essen (AöR), West-German Cancer Center Essen, Hufelandstraße 55, 45147 Essen, Germany

**Keywords:** Renal cell carcinoma, Medical therapy, Checkpoint inhibitor, Tyrosine kinase inhibitor, Guideline

## Abstract

**Purpose:**

The treatment landscape in metastatic renal cell carcinoma (mRCC) has evolved dramatically in recent years. Within the German guideline committee for RCC we evaluated current medical treatments and gave recommendations.

**Methods:**

A systematic review of published evidence for medical treatment of mRCC was performed (July 2016–August 2019) to cover the duration from last guideline update in 2016. Evidence was graded according to SIGN (http://www.sign.ac.uk/pdf/sign50.pdf). Recommendations were made on the basis of a nominal group work with consensus approach and included patient advocates and shareholder of the German RCC treatment landscape. Each recommendation was graded according to its strength as strong recommendation (A) or recommendation (B). Expert statements were given, where appropriate.

**Results:**

Strong first-line recommendations (IA) exist for axitinib + pembrolizumab (all risk categories) and ipilimumab + nivolumab (intermediate or poor risk only). Axitinib + avelumab is a recommended first-line treatment across patients with any risk category (IB). In patients who are not candidates for immune check point inhibitor (ICI) combinations, targeted agents should be offered as an alternative treatment. Subsequent treatment after ICI-based combinations remain ill-defined and no standard of care can be formulated.

**Conclusion:**

ICI-based combinations are the first-line standard of care and should be considered accordingly. There is an unmet medical need for pivotal studies that define novel standards in patients with failure of ICI-based combinations.

## Introduction

The landscape for systemic therapy in metastatic renal cell carcinoma (mRCC) has changed dramatically in recent years. Today, tyrosine kinase inhibitors (TKI), inhibitors of the mammalian target of rapamycin (mTORi) and immune checkpoint inhibitors (ICI) as single agent or combination therapies built the backbone of medical treatment. As a tribute to the rapid development novel combinations with 3^rd^ generation TKI have reported positive results and are 1st line candidates for approval. These trials reported after the search period for our systematic review and are not subject for consideration. Our analysis was performed with the intention to build an evidence-based approach for agents currently licensed by the European Medicines Agency (EMA) at the time of literature search. The German version of treatment recommendations can be accessed online.^1^

### First-line therapy in clear cell RCC

Treatment recommendations are based on the International Metastatic RCC Database Consortium (IMDC) risk categories [1]. Individual disease parameters are subjective and therefore will not be discussed in this article. However, they are considered appropriate for individualization of treatment options in the clinic. Given the existing differences among these first line studies, advantages and disadvantages of a given treatment should be weighed in the context of the therapeutic aim (such as long-term outcome or symptom control) for individualized treatment selection.

CM214 was the first immune combination (ipilimumab + nivolumab) that reported improved clinical outcome parameters when compared with sunitinib, which is the trial with the most mature data today. However, the primary endpoint was based on patients with at least 1 risk factor only and reflects therefore a selection of patients with worse prognosis. In contrary, JR101 and KN426 trials considered all risk categories for their primary analysis appropriate and compared axitinib with either avelumab or pembrolizumab to sunitinib. These three trials are considered milestones in the development of ICI-based combinations in mRCC and their key parameter are listed in Table 1. Differences between trial populations exist and do not warrant direct comparison between trials. Therefore, Table 1 should be considered as reference of key parameters of these trial only.

**Table 1:** Key clinical parameter of first line trials with licensed agents

	KN426 [2, 10] Axitinib + pembrolizumab	CM214 [3, 5] Ipilimumab + nivolumab	JR101 [4, 11] Axitinib + avelumab
N	861	1096	866
IMDC risk (% of patients)			
Favourable	32	23	21
Intermediate	55	61	61
Poor	13	17	16
Median follow-up (mo.)	30.6	55.0	19.3
Objective response rate (%)	60.2	39.1	52.5
PFS median (mo) (HR; 95%CI)	15.4 vs. 11.1 0.71 (0.60–0.84)	12.2 vs. 12.3 0.89 (0.76–1.05)	13.3 vs. 8.0 0.69 (0.57–0.83)
OS median (mo) (HR; 95%CI)	NR vs. 35.7 0.68 (0.55–0.85) *P* = 0.0003	NR vs. 38.4 0.69 (0.59–081)	NR vs. NR 0.8 (0.62–1.03) *P* = 0.04^a^
Discontinuation rate	30.5% 1 agent 10.7% both agents	22%	18.4% 1 agent 3.5% both agents
Dose-reductions	20.3%	0%	42.2%
Grade ≥ 3 adverse events	75.8%	46%	71.2

^a^Did not meet pre-specified boundary for significance

Based on these findings, recommendations considered axitinib + pembrolizumab and ipilimumab + nivolumab standard first-line options (both IA recommendations), which showed OS benefit (Table 2) [2, 3]. Axitinib + avelumab is considered as another option, but the pivotal trial did not report a statistically significant OS benefit in the interim analysis (Table 2) [4]. The principle activity of this regimen supports its use, but the lack of OS advantage has led to a IB recommendation.

**Table 2:** First line options which are considered standard of care

Recommendations	Level of Evidence (LoE)	Grade of recommendation	Grade of consensus
Offer axitinib + pembrolizumab to treatment-naïve patients with any IMDC-risk category	1 −	A	Strong
Physicians may offer axitinib + avelumab to treatment-naïve patients with any IMDC-risk category	1 −	B	Consensus
Offer ipilimumab + nivolumab to treatment-naïve patients with intermediate or poor IMDC-risk category	1 −	A	Strong

The choice between treatments should take the pros and cons of each option into account and also consider patient and tumor parameters to individualize therapy to the needs of a patient. A therapeutic aim should be formulated and the most appropriate regimen chosen. For instance, high tumor burden or critical anatomical location as well as symptomatic disease have a short-term aim of disease- and symptom-control. This may favor regimens with the highest chance for response, such as axitinib + avelumab (ORR: 52.5%) or pembrolizumab (ORR: 60.2%) [2, 4]. On the contrary, ipilimumab + nivolumab has reported favorable long-term results, with a median follow-up of 55 months, indicating durable responses for this TKI-free regimen [5]. Differences in the tolerability profile exist between regimen (Table 1), which may contribute to differences in health-related quality of life (HR-QoL) or symptom scale measures. Axitinib-based combinations require chronic TKI exposure, these agents did not report benefit in patient reported outcomes (PRO) [6]. On the contrary, the TKI-free combination of ipilimumab + nivolumab was associated with an improvement in FKSI-19 (total score), when compared to sunitinib [7].

More recently, 3^rd^ generation TKI reported positive phase III trials in first-line treatment. Cabozantinib + nivolumab reported a HR for OS of 0.60 (98.9%CI 0.40–0.89; *P* = 0.001) and was recently approved by EMA as a novel first line option [8]. Moreover, Lenvatinib + pembrolizumab achieved a HR for OS of 0.66 (95%CI 0.49–0.88; *P* = 0.005), thereby underscoring the relevance of the 3^rd^ generation TKI as combination partners [9]. These agents will be formally discussed within the next update of the German guideline.

Patients who are not candidates for ICI combinations should receive a VEGF targeted therapy as an alternative treatment. Based on previous studies in mRCC a differential recommendation for VEGF targeting agents exists. IMDC risk categories were used to categorize these recommendations and are depicted in Table 3. While these VEGF targeting agents received a stronger recommendation in patients with favorable risk (IA), they may also be offered in patients with at least one risk factor (IB).

**Table 3:** First line recommendations for patients who are not candidates for ICI combinations

Recommendations	Level of Evidence (LoE)	Grade of recommendation	Grade of consensus
In patients who are not candidates for check point inhibitor therapies, offer alternative treatment according to IMDC risk:			
Favourable: bevacizumab + interferon, pazopanib, sunitinib or tivozanib	1+ +	A	Strong
Intermediate: cabozantinib, pazopanib, sunitinib or tivozanib	1+ + /1 −	B	Strong
Poor: cabozantinib or sunitinib	1+ /1 −	B	Consensus

### Treatment of patients with favorable risk

Ipilimumab + nivolumab was not superior to sunitinib treatment neither in regard to efficacy parameters nor OS and is therefore not recommended in favorable risk patients (Tables 2 and 4) [5]. Treatment choices consider ICI combinations as the mainstay of therapy, which may consist of axitinib + pembrolizumab or avelumab (Table 2). For both pivotal trials data remains still immature for the subgroup of patients with a favorable risk profile. In these patients, a higher anti-tumor activity was detected compared to sunitinib, but no significant OS benefit was noted between groups (Table 4) [10, 11]. Hence, overall superiority for ICI combinations over sunitinib could not be shown in this group of patients. However, the low event rate renders this analysis immature and no conclusive statement in regard to OS can be given. In spite of these limitations, single agent TKI treatment may be offered to patients who are not candidates for surveillance and do not require the higher chance for response or PFS advantage, which is associated with axitinib-based combinations.

**Table 4:** Post-hoc subgroup analyses according to IMDC risk categories

	IMDC risk (% of patients)	Favourable	Intermediate	Poor
Ipilimumab + nivolumab [3, 5]	Objective response rate (%)	29.6	41.9	
	PFS HR (95%CI)	1.84 (1.29–2.62)	0.74 (0.62–0.88)	
	OS, HR (95%CI)	0.93 (0.62–1.40)	0.65 (0.54–0.78)	
Axitinib + pembrolizumab [2, 10]	Objective response rate (%)	69.6	55.8	
	PFS HR (95%CI)	0.79 (0.57–1.09)	0.69 (0.56–0.84)	
	OS, HR (95%CI)	1.06 (0.60–1.86)	0.63 (0.50–0.81)	
Axitinib + avelumab [4, 11]	Objective response rate (%)	67.0	53.1	31.9
	PFS HR (95%CI)	0.626 (0.397–0.986)	0.756 (0.603–0.948)	0.514 (0.342–0.774)
	OS, HR (95%CI)	0.812 (0.336–1.960)	0.860 (0.615–1.202)	0.570 (0.363–0.895)

### Treatment of patients with intermediate or poor risk

Patients with at least 1 risk factor derive a substantial treatment benefit from ICI-based combinations and reflect therefore the standard of care (Table 2). Superiority for overall survival was consistent across all ICI-combinations (Table 4) although effect size in efficacy parameter varied between studies. The choice between ipilimumab + nivolumab, axitinib + pembrolizumab or avelumab should be made according to individual patient parameters, as outlined above.

### Second line therapy in clear cell RCC

A high level of evidence exists after the failure of monotherapy with a TKI. In this uncommon clinical scenario, cabozantinib or nivolumab are considered standards of care and receive the strongest recommendation (IA) (Table 5) [12, 13]. Lenvatinib + everolimus has randomized phase II data supporting its use after TKI failure, which renders it a treatment option but with a weaker recommendation (IB) (Table 5) [14].

**Table 5:** Recommendations for second line therapies after single agent VEGF-targeted therapy

Recommendations	Level of Evidence (LoE)	Grade of recommendation	Grade of consensus
After failure of monotherapy with VEGF-targeted therapy, offer:			
Cabozantinib or nivolumab	1 −	A	Strong
Lenvatinib + everolimus	1 −	B	Strong

With the rapidly changing first-line landscape, data remains scarce for subsequent therapies after ICI-based combinations. The paucity of randomized clinical data in this treatment scenario was prohibitive to formulate a standard of care. Instead, statements of expert opinions were formulated to guide the treatment choice. Given the principle activity of TKIs after ICI-based therapies in retrospective series, a TKI-based therapy was considered appropriate after ipilimumab + nivolumab or axitinib + pembrolizumab or avelumab (Table 6) [15, 16]. In the rare event that first-line therapy consisted of an mTOR inhibitor, either a TKI-or based therapy or nivolumab are considered appropriate (Table 6).

**Table 6:** No standard of care exists after failure of ICI-based combinations

Statement	Level of Evidence (LoE)	Grade of consensus
After failure of check point inhibitor therapy:		
Is no standard of care defined	Expert opinion	Strong
A TKI-based therapy should be given	Expert opinion	Consensus
After failure of axitinib + avelumab or pembrolizumab, offer a TKI-based therapy	Expert opinion	Strong
After failure of mTOR inhibitor therapy:		
Is no standard of care defined	Expert opinion	Strong
Either TKI-based therapy or nivolumab should be given	Expert opinion	Strong

### Third line therapy

There is no standard of care defined and no specific recommendation can be given. Given the differences in the mechanism of action or kinase inhibitory profile, the subsequent therapy should consist of an agent that has not been used in previous therapies (Table 7).

**Table 7:** Third line therapy

Statement	Level of Evidence (LoE)	Grade of consensus
There is no standard of care in 3rd line therapy defined	Expert opinion	Strong
Choice of subsequent therapy should be guided by previous exposure and should implement a different agent	Expert opinion	Strong

### Non-clear cell renal cell carcinomas

Non-clear cell histologies resemble a heterogenous group of patients and they account for about 20–25% of RCC [17]. Today, there is an increasing body of evidence that supports the classification of these tumors as distinct entities. The molecular diversity of these cancers paved the way for a differential drug development and renders the summation of these tumors as one heterogenous group of RCCs obsolete. However, clinical data remains scarce and the current guideline version does not include specific recommendations for these rare entities. This section is subject for recommendations in future versions of our guideline.

Of note, the term sarcomatoid RCC is frequently used, but this does not represent a classified group of RCC. Instead it is an advert biologic feature, which may occur in any RCC entity [18]. Clinical data for medical treatment of this subgroup is mainly derived from randomized clinical trials, which included ccRCC only. Hence, the majority of clinical data for treatment represent ccRCC with a sarcomatoid component, which should be differentiated from other sarcomatoid RCC (non-clear) entities. Furthermore, pure mesenchymal renal tumors represent a distinct class of renal malignancies and should be reported and treated accordingly.

## Data Availability

NA.

## Footnotes

1 https://www.awmf.org/uploads/tx_szleitlinien/043-017OL-l_S3_Diagnostik-Therapie-Nachsorge-Nierenzellkarzinom_2020-09_1.pdf.

## Code availability

NA.
